# Physical activity and functional social support in community-dwelling older adults: a scoping review

**DOI:** 10.1186/s12889-024-18863-6

**Published:** 2024-05-20

**Authors:** Paula Steinhoff, Amelie Reiner

**Affiliations:** Institute of Sociology and Social Psychology, University of Cologne, Cologne, Germany

**Keywords:** Physical activity, Social support, Older adults, Scoping review

## Abstract

**Background:**

Globally, the population of older people is increasing and is estimated to reach nearly 2.1 billion by 2050. Physical activity (PA) is one of the key components for successful ageing. However, PA decreases with age and many older adults do not meet PA guidelines. Previous research has shown that social support (SOSU) is related to PA in older people. The aim of this scoping review is to identify and map all of the available evidence and to explore the association between functional SOSU and PA in older adults. Functional SOSU consists of emotional, informational, instrumental and companionship SOSU and social comparison.

**Methods:**

A scoping review was conducted using the Joanna-Briggs manual. Quantitative and qualitative studies investigating associations between functional SOSU and PA levels in older adults (mean age ≥ 60 years) were identified through a systematic search in seven electronic databases up to August 2023. After removing duplicates, 20,907 articles were screened for titles and abstracts. The results were analysed separately for different types of SOSU.

**Results:**

116 articles met the inclusion criteria; 72% were quantitative studies and 28% were qualitative studies. Most studies used self-reported PA measures, only 23% of the studies used objective-reported PA measures. Most studies of SOSU for PA reported positive associations but the evidence is inconclusive when the source of support is considered. PA is positively associated with general, emotional, informational, and companionship SOSU, while instrumental support may occasionally be negatively associated. Companionship support is particularly influential on PA, especially in group settings, as it promotes social connectedness. Qualitative studies show that social comparison also supports PA. Different forms of SOSU generally show positive associations with PA.

**Conclusions:**

While the evidence on the association between functional SOSU and PA is mixed, most studies show that there is a positive association. PA may also be a strategy for improving social contact and social integration. This study offers a comprehensive overview of measures for SOSU and PA and thereby informs future research and policy-making.

**Supplementary Information:**

The online version contains supplementary material available at 10.1186/s12889-024-18863-6.

## Introduction

Regular physical activity (PA) is a key factor in preventing and managing non-communicable diseases and promoting mental health, cognitive functioning and quality of life [1, 2]. PA is defined as “any bodily movement produced by skeletal muscles that requires energy expenditure” [3]. It encompasses exercise, sport and physical activities performed as part of daily living, occupation, leisure or active transportation. People who engage in recommended levels of PA have a 20–30% lower risk of dying prematurely [3]. Physical inactivity is nowadays considered a pandemic [4]. It is the fourth leading cause of death worldwide [5]. It is estimated that physical inactivity accounts for 6–10% of the incidence of major non-communicable diseases such as cardiovascular diseases, diabetes mellitus type 2 and some types of cancers [6] and for 7–8% of depression and dementia [3]. This preventable burden not only affects mortality and morbidity [6] but also the economy [4] of nations worldwide. More than 80% of adolescents and more than one in four adults do not engage in the WHO’s recommended levels of PA [3].

Regular PA is even more important for older adults. The global population of older people is increasing and is projected to reach 1.4 billion by 2030 [7]. In most countries, however, PA decreases with age [3], with around 67% of people aged 65–74 and 75% of people over 74 not meeting recommendations [8]. Nonetheless, older adults benefit particularly from regular PA to preserve their physical, social and mental health, delay dementia and prevent falls [3]. Older adults should perform a total of at least 150–300 min of moderate-intensity aerobic PA or 75–150 min of vigorous-intensity aerobic PA each week or an equivalent combination of both [9]. Research has shown that older adults who receive more social support (SOSU) from family or friends are more physically active [8]. Furthermore, there is a negative association between social isolation and loneliness and PA in middle-aged (50–64) and older people (65 or older) [10]. People of this age group are at higher risk of feeling lonely, having decreased SOSU and engaging in lower levels of PA due to declining physical capacities [8, 11].

While much evidence exists of the interplay between PA and SOSU, there has been no systematic overview of studies addressing this topic in older adults. One difficulty in the literature is that alternative terms such as social network, loneliness, social isolation and social integration are used interchangeably, even though these concepts all measure different phenomena. Functional SOSU is one dimension of SOSU and is a multidimensional concept.

It suggests that social relationships offer various types of supportive functions and these functions may be more or less effective in responding to specific problems or situations. Willis and Shinar [12] identify five dimensions of functional SOSU. Emotional support relates to the availability of someone who listens, cares and accepts the individual and provides encouragement, esteem and reassurance. Instrumental support is more tangible and refers to the provision of practical assistance where needed, such as by helping with transportation, assisting in the home or lending money. Informational support entails providing valuable knowledge to address problems: this includes providing information about resources and services as well as offering advice and guidance. Companionship support encompasses the presence of individuals with whom one can partake in social and leisure activities. The final dimension, often referred to as validation, feedback or social comparison, is rooted in the idea that social relationships can offer insights into the appropriateness or conformity of behaviours. In line with this framework, this scoping review analyses the sub-dimensions of functional SOSU, namely emotional, instrumental, informational and companionship support and validation. Studying the functional aspects of SOSU is important for exploring PA among older adults. This is because each dimension offers different types of SOSU that can be important for PA adherence in older adults. For instance, emotional SOSU could provide encouragement to start exercising, while instrumental SOSU could involve driving someone to exercise classes. Informational SOSU could entail providing safe instructions for exercising, and companionship SOSU could involve going on a walk together. Validation could be gained from seeing peers engage in PA and becoming motivated to do so as well. This approach has been used in a previous systematic review and meta-analysis concerning SOSU and PA in adolescent girls [13].

Lindsay-Smith et al. conducted a comprehensive systematic review on the association between PA, loneliness and SOSU [8]. However, their literature search ended in August 2014 and the intervening period, especially due to the COVID-19 pandemic, has seen important research around this topic. Importantly, as the authors conducted a systematic review, qualitative studies were not considered. The aim of this scoping review is to both update and deepen the systematic review [8] by identifying and mapping all of the available evidence and to explore the association between functional SOSU and PA in older people.

By including quantitative, qualitative and mixed methods studies, this review aims to inform future research by offering a comprehensive overview of measures for functional aspects of SOSU and subjective and objective PA in older adults. Particularly through its overview of qualitative studies, it identifies facilitators and barriers for older adults to engage in PA and helps build a deeper understanding of why older adults do not exercise. In addition, it informs policy-makers by presenting the available evidence on why older adults do not participate sufficiently in PA.

## Methods

A scoping review was conducted in accordance with the Joanna Briggs Institute Manual [14]. This review was pre-registered. The review protocol can be accessed at https://osf.io/9uhgb. A scoping review differs from a standard systematic review in that it does not attempt to synthesise the evidence but rather serves to determine the extent and nature of the evidence. It identifies gaps and clarifies concepts in the literature and makes recommendations for future research and policy-making [15–17]. Within this topic, a scoping review is particularly important as there are no clear concepts or definitions of the term ‘social support’. This paper focuses on the functional aspects of SOSU, namely emotional, instrumental, informational and companionship support and validation [12]. Hence, it gives a comprehensive overview of measurements of functional aspects of SOSU.

We followed the nine steps proposed by the JBI for conducting scoping reviews [14]. The literature search was conducted up to the 8th of August 2023. Various search terms were developed (Supplementary material 1) for each of the three dimensions of the research aim (‘older adults’, ‘social support’ and ‘physical activity’). The development of the search strategy followed a three-stream approach: first of all, we checked for search terms in prior published reviews; after this, we checked for MeSH terms in PubMed; subsequently, an interdisciplinary team of researchers met to discuss further search terms (PS, health scientist; AR, LE and KH, sociologists with a focus on health and ageing). We searched the databases PubMed, Web of Science, Scopus, APA PsycInfo, ProQuest, SocINDEX and PSYNDEX and in the Cochrane Library. In addition, we checked literature reference lists from other reviews to identify further relevant literature. English peer-reviewed empirical studies that addressed the association between functional SOSU and PA in an older population were included. We did not apply limits in terms of time or geographic location. We included studies in which the minimum age of participants was 40 years but the mean age of participants was at least 60 years; a minimum age of 40 years is in line with the common ageing surveys. We excluded editorials, comments, study protocols, conference proceedings, grey literature and literature reviews. Furthermore, we excluded studies which addressed only a specific patient group or concerned only institutionalised or hospitalised individuals [8, 18]. Since this is a scoping review, no critical appraisal or risk of bias assessment was conducted [19]. We identified a total of 35,777 articles in our initial search. After the removal of duplicates, 20,907 abstracts were screened for eligibility. This screening process was carried out independently by two researchers (PS and AR), who remained blinded throughout. In cases where they could not reach a decision, a third reviewer (LE) was consulted and a final decision made after thorough discussion. To ensure reliability, PS and AR conducted two pilot screenings.

## Results

Subsequently, 255 full texts were read, and 116 articles were included in the final analysis. These were published between 1989 and 2023, with over 50% published later than 2017.

**Fig. 1:**
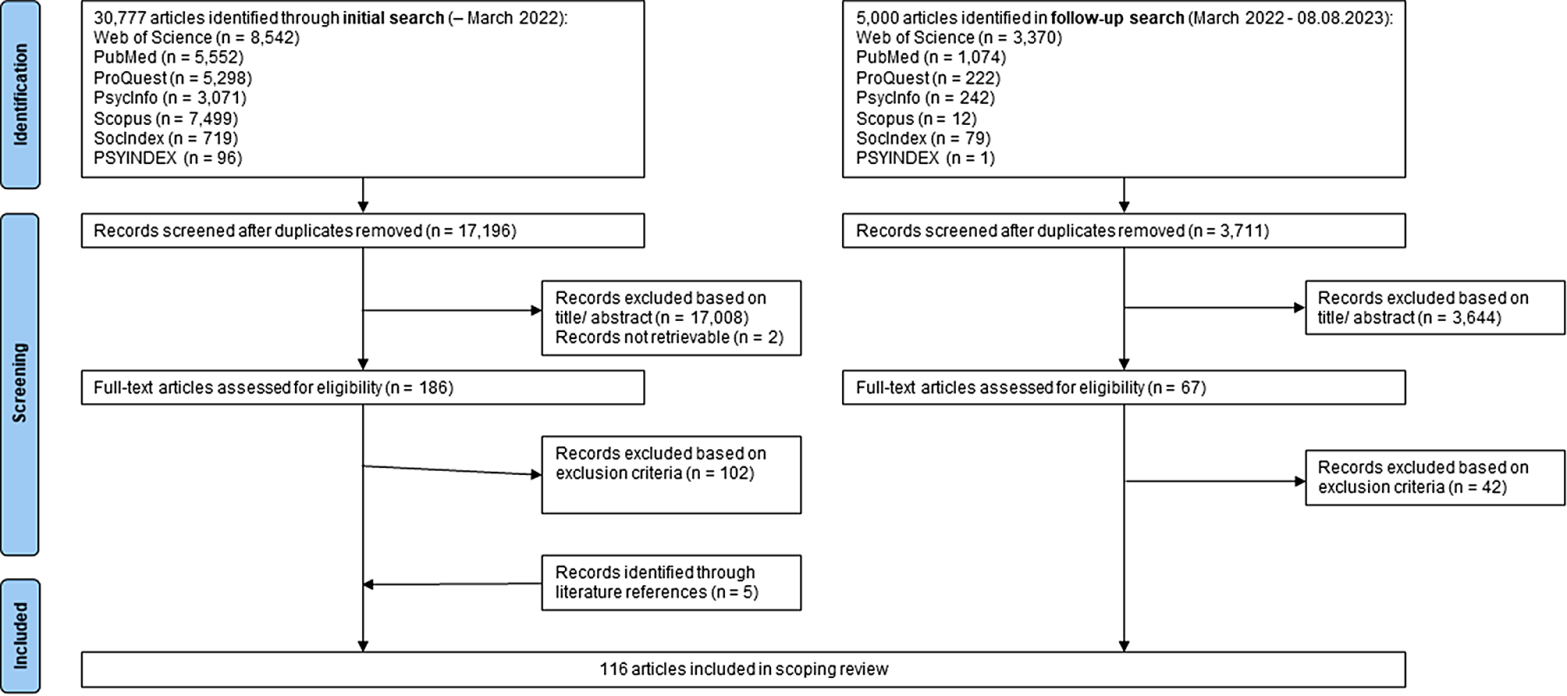
Adapted PRISMA-P flowchart

The entire process is visually represented in the adapted PRISMA-P flowchart (Fig. 1 adapted PRISMA-P flowchart).

This paper provides a descriptive overview of the results of each of these studies, including the different measures for PA and SOSU in a data extraction table (Supplementary material 2). This table was completed independently by two researchers (PS and MW). Approximately half of the studies included (48%) were conducted in North America, with 44 (38%) conducted in the USA. A further 26% were conducted in Asia and 16% in Europe. Results were broadly stable across geographic areas. Eight articles focused on ethnical minority groups in the USA [20–24], the UK [25] and Australia [26] and four studies compared different ethnical populations which each other [27–30]. In terms of gender, 22 articles (19%) concerned only women and two only men. Over 70% of the included studies were quantitative. Approximately 56% (*n* = 65) were cross-sectional studies, 5% (*n* = 6) were longitudinal studies and 11% (*n* = 13) were intervention studies. Of the total, 6% (*n* = 7) used a mixed methods design, while 22% (*n* = 25) used a qualitative research design. Quantitative studies varied considerably in terms of sample size (from *n* = 34 to *n* = 78,002) and measurement quality (from one item per variable to validated scales). Of the quantitative studies analysed, 97% (*n* = 83) controlled for age or gender, and 77% (*n* = 66) controlled for both. Qualitative and some mixed methods studies lacked standardised quality criteria such as sample size or measurement instruments.

The included papers covered a wide range of research topics, but all explored aspects of functional SOSU in older adults. Among the quantitative studies, most analysed either general SOSU (*n* = 26) or SOSU for PA (*n* = 33). There was also considerable variation in the assessment of PA. Of the quantitative studies, 77% relied solely on subjective measures of PA. In 14%, only objective measures were used, while 9% used a combination of objective and subjective measures. Objective PA was mainly measured using device-based methods (accelerometer; *n* = 15) or attendance records (*n* = 4). Subjective PA was mostly measured using single items, although some studies used validated scales, such as versions of the IPAQ (*n* = 12). In addition, 24% of the studies explicitly focused on PA in older adults, using either the Community Healthy Activities Model Program for Seniors (CHAMPS, *n* = 7) or the Physical Activity Scale for the Elderly PASE, *n* = 14).

Overall, 65 of the 86 quantitative studies (76%) found at least partial evidence of a positive and significant association between SOSU and PA. Four studies reported both positive and negative associations [31–34], while four studies found a negative association between SOSU and PA [24, 35–37]. In terms of sources of SOSU, the evidence is inconclusive. Seven quantitative studies [23, 28, 66, 68, 70, 83, 87] found that friends were more important, while four studies found that family SOSU was more important [65, 71, 84, 92]. All qualitative studies described a positive relationship between SOSU and PA. The results are presented separately for the different types of functional SOSU.

### General social support

Numerous studies have examined the association between general SOSU and PA in older adults, but the methods used to measure SOSU vary. Some studies have used instruments such as the DSSI [33, 38, 39], the MSPSS [36, 40, 41] or the LSNS [23, 42, 43], while most measure general SOSU with very few items, both of which make it difficult to compare results. Nonetheless, despite these measurement differences, the majority of studies consistently show a significant positive relationship between general SOSU and PA in older adults. This is true not only for studies that measure PA subjectively but also for those that use objective measures [44, 45].

These positive associations have been found in various countries, including Malaysia [33, 39], China [46–48], South Korea [40, 49], Ghana [50], Jamaica [51], Canada [34, 52], the USA [23, 43, 44, 53–56], Sweden [45], Spain [38] and Germany [57]. One study reported gender-specific effects, with SOSU showing a positive association with PA in Canadian women but not in men [52]. In a study of older Samoan women in the USA, Levy-Storms and Lubben found a positive association between SOSU from a non-kin network and PA levels, but no significant association between kin-based SOSU and PA [23]. Marthammuthu et al. found mixed results in Malaysian women: on the one hand, women with an increase in social interaction had significantly higher levels of PA; on the other hand, an increase in subjective SOSU was negatively associated with PA [33]. A study that divided SOSU into objective support, subjective support and support use found that objective SOSU and support use were significantly associated with PA, whereas subjective SOSU and PA did not show a significant relationship in older Chinese [47]. Kumar et al. found that a lack of exercise was significantly associated with a lack of general SOSU in India [42]. An intervention study conducted in the USA found that higher SOSU from family and friends positively predicted increased PA levels in a telephone-based PA programme for older adults [56]. However, there were exceptions to this general trend, with a few studies reporting no significant association between SOSU and PA in older adults [58–60].

### Functional social support specific for physical activity

SOSU for PA covers functional aspects of SOSU that are specifically relevant to PA. Most studies that have specifically examined SOSU for PA have found a positive correlation between SOSU for PA and actual levels of PA. Sixteen studies [20, 21, 27, 30, 31, 37, 61–70] used the Social Support for Exercise Scale from (SSES), which covers companionship, informational, emotional and instrumental support for exercise, to assess SOSU for PA. Not all studies using the SSES have produced consistent results. For example, Gothe found no effect of SOSU on objective or subjective PA in older African Americans [21] and Hall and McAuley found no association between SOSU and objective PA in older US-American women [64]. A 12-week intervention study of a community-based programme to promote PA found that while the intervention group experienced greater improvements in exercise-related SOSU from friends, both the control and intervention groups experienced decreases in subjective and objective PA [37]. Brassington et al. [61] evaluated a 12-month exercise intervention study in the USA in which participants received regular telephone exercise advice from a trained health professional. Exercise-related SOSU showed no association with longer-term adherence. Gellert et al. [31] conducted an intervention study in Germany comparing singles, participants who exercised with their partner, and partnered participants who did not. At baseline, there was no difference in PA levels between the three groups. At follow-up, couples who participated in the intervention showed a positive correlation between SOSU and PA, with SOSU increasing in this group. For singles and those whose partners chose not to participate, SOSU and PA were negatively associated.

Other studies used different scales to measure SOSU for PA. In a 6-month longitudinal study, Warner et al. found that friends’ SOSU at baseline predicted PA at the 6-month follow-up [70]. Family support was significantly positively associated with leisure walking in Brazil [71]. In a longitudinal study, Smith et al. observed a positive relationship between SOSU for PA and PA over time in older Australians [72]. Rhodes et al. examined adherence to a strength training programme in older Canadian women over the first 6 months. SOSU for PA was positively associated with participation after 3 months, but not after 6 months [73]. Purath et al. investigated the relationship between SOSU and PA in 34 older adults in the USA: they found a significant positive association only between SOSU from healthcare providers and PA and no significant association for support from peers [74]. One intervention study in the USA showed positive effects of SOSU interventions on PA levels [75], and general SOSU for PA was positively associated with PA [76, 77]. Emotional and informational support for PA was positively associated with exercise in older US-American adults [78]. O’Brien Cousins [79, 80] and Wilcox et al. [30] both found a positive association between SOSU for PA and PA in women. Focus groups highlighted the importance of SOSU for PA as a facilitator for older adults to engage in group exercise [81] and for older women to engage in strength training [27]. In general, most studies support a significant positive association between SOSU for PA and actual levels of PA [82]. However, evidence as to the most effective source of support is mixed, with some studies favouring support from friends [27, 66, 68, 83] and others favouring family support [65, 84].

### Emotional social support

Numerous studies have focused on the relationship between emotional SOSU and PA in older adults. Four cross-sectional studies [85–88] and one longitudinal study [89] found a positive association. However, the results are highly variable and may also depend on the type of PA studied. Malek Rivan et al. found no significant association between emotional SOSU and PA in older Malaysians [35]. Perrino et al. found no significant association between emotional SOSU and walking in Hispanic-Americans [24], while Komazawa et al. found no significant association between emotional SOSU and exercise frequency in a longitudinal study in older Japanese adults [90]. Yamakita et al. investigated the role of perceived and given emotional SOSU and participation in exercise groups in 78,002 older adults in Japan. Both perceived and given emotional SOSU were significantly associated with participation in sports groups [91]. Loprinzi and Joyner assessed the source of emotional SOSU, asking older adult participants in the USA whether they received emotional SOSU from spouses, children, siblings, neighbours, church members or friends and assessing their subjective PA. Only emotional SOSU from friends was associated with meeting PA recommendations in older US-Americans [87]. In 2017, Loprinzi and Crush examined the sources of emotional SOSU among the same range of social ties but with objective PA. They found a positive association only between spousal emotional SOSU and PA [92]. While it is evident that emotional SOSU is associated with PA, the evidence around the source of SOSU is not consistent.

Qualitative studies [22, 25, 81, 93–99] and a mixed methods study [100] have also highlighted the importance of emotional SOSU. Huffman et al. highlighted its role in re-engagement and maintenance of PA in the USA [93], while Wahlich et al. found that the presence of friends and family was crucial in motivating older adults to engage in PA by providing encouragement and support to try new exercises in the UK [99].

### Informational social support

No quantitative studies have specifically examined the association between informational SOSU and general PA. Perrino et al. investigated the relationship between informational SOSU and walking in older Hispanic-Americans but found no significant association [24]. Nevertheless, the qualitative components of three mixed methods studies [97, 100, 101] and five qualitative studies [22, 29, 93, 95, 96] highlight the importance of informational SOSU for PA and exercise.

Arnautovska et al. found that informational SOSU from healthcare professionals played an important role in facilitating PA in older Australians [101]. Similarly, Gothe and Kendall found that informational support from physicians was important for PA in older African-American women [22]. In line with this, Leung et al. found that professional guidance encouraged older adults to walk more in older adults in Hongkong [95]. Oliveira et al. highlighted that informational SOSU from friends, family and trainers served as an important motivator for exercise and class attendance in older Australians [97]. Gagliardi et al. evaluated a volunteer programme in Italy and found that ‘socialisation’ was an important reason for participation, with information exchange and peer support highly valued. Participants particularly valued learning from each other [100]. Huffman and Amireault also emphasised the importance of sustained PA engagement through informational SOSU in older US-Americans [93]. Marthammuthu et al. identified informational SOSU as a key factor in encouraging exercise among older rural Malaysian women [96]. Mathews et al. identified a lack of knowledge about safe exercise as an important barrier, with informational SOSU specific to older people as an enabling factor for PA [29]. Similarly, Beselt et al. found that older women living alone in Canada particularly valued group PA programmes, especially because the trainer’s instructions provided a sense of security [102].

### Instrumental social support

Limited research has examined instrumental SOSU in relation to PA, and the evidence is mixed. For example, Zimmer and McDonough found a significant negative association between instrumental SOSU and PA among Canadian older adults not living alone, but not among those living alone [34]. Malek Rivan et al. also found a negative relationship between instrumental SOSU and PA [35]. Perrino et al. observed that higher levels of instrumental SOSU were associated with lower levels of walking in older Hispanic-Americans [24]. In a longitudinal analysis of older Japanese adults, Komazawa et al. reported that instrumental SOSU was negatively correlated with PA in financially stressed women, but not in men [90]. In contrast, Loprinzi and Joyner found a positive association between financial SOSU and PA levels [87]. In line with this, Choi et al. found that financial SOSU was an important facilitator of exercise in South Korea. Lack of financial resources was an important barrier to PA [103]. Yamakita et al. examined instrumental SOSU given and received and found that both were associated with sports group participation in older Japanese adults [91].

Qualitative studies offer a more positive perspective on the relationship between instrumental SOSU and PA. Janevic and Connell examined support and exercise in female US-American carers and found that instrumental SOSU, such as having someone to look after their care-receiver, facilitated exercise, whereas a lack of support hindered it [104]. Marthammuthu et al. identified logistical problems in attending exercise programmes as a major barrier for older Malaysian women [96]. Jones et al. highlighted the crucial role of instrumental SOSU in supporting exercise among older retired US-American women [94], and Arnautovska et al. also highlighted its importance in facilitating PA in older Australians [101].

### Companionship social support

Companionship SOSU is an important determinant of PA in older adults. Ory et al. and Shores et al. found a positive association between companionship SOSU and PA [105, 106]. Salvador et al. investigated the association between PA and SOSU for exercising, walking or cycling with friends, neighbours or relatives in Brazil and found a significant positive association for men but not for women [107]. However, Wagner et al. found no significant association between park-based PA and companionship SOSU in a study of older adults in China and Germany [108]. McAuley et al. attempted to predict the long-term exercise behaviour of US-American older adults after a 6-month RCT with an 18-month follow-up. They found no direct effect of exercise frequency or companionship SOSU from the exercise group on long-term activity rates [109]. Thomas et al. found a positive significant association between a buddy peer support intervention and subjective PA in older Chinese [110]. Cai et al. analysed the relationship between a similar intervention and objective PA in older Chinese and found a positive association [111]. Crist et al. examined the relationship between a peer-led group PA intervention and objective PA in older US-Americans. They found a significant increase in PA in the intervention group at 12 months, and participants maintained the increase over 2 years [112]. Chia et al. found a significant positive association between companionship SOSU and objective PA in Taiwanese men and women, but the association was stronger in women [113]. Huang et al. found that walking together in a group significantly increased objective PA in older Taiwanese men [114].

Numerous mixed methods [67, 97, 100] and qualitative studies [81, 94, 95, 99, 102–104, 108, 115–122] have also highlighted the importance of companionship SOSU for PA. These studies underline the positive effects of group exercise, friendship formation, being part of a set group, and increased social interaction as facilitators of PA engagement.

Research suggests that companionship SOSU and PA have a close and bidirectional relationship. Specifically, engaging in PA in a group setting appears to be a critical factor in encouraging older adults to participate in regular exercise [22, 25, 81, 94, 97, 100, 102, 103, 115–117, 119, 120, 123]. In addition to promoting PA, companionship SOSU also serves as an essential means of maintaining social connections for older adults, thereby improving their overall well-being. Furthermore, engaging in PA with others provides a sense of safety and security [81, 95, 96, 102].

### Validation

There are no quantitative studies that focus on the aspect of validation, and only six qualitative studies have addressed this issue. Gagliardi et al. conducted a mixed methods study to evaluate an intervention programme for older adults in Italy. Participants reported that the opportunity to compare themselves with peers was an important reason for participating in the programme [100]. Similarly, Huffman et al. found that feedback, particularly from trainers, was an important facilitator of overall PA maintenance [93]. Zhang et al. explored factors influencing PA participation among older adults in the UK. Participants highlighted the importance of social comparison with peers’ health and PA behaviours as a motivating factor to engage in PA [124]. The perspectives of older women living alone in Canada who participated in group exercise were explored by Beselt et al. Some participants emphasised the motivational value of comparing themselves with others in the group. Seeing other active women was particularly uplifting, and observing those who were less physically fit served to inspire participants to either maintain or increase their own levels of PA [102]. When examining the relationship between SOSU and Canadian group PA programmes, Zimmer et al. found similar results. Giving and receiving feedback from others was an important motivator for participating in a group programme, while seeing others performing exercises motivated participants to try them themselves [81]. Patterson et al. similarly found that social comparison with other participants increased motivation for attending a group PA programme in older Canadian women [98].

### Other social support

Numerous studies have explored alternative aspects of SOSU and its association with PA in older adults. Mowen et al. examined SOSU network size and satisfaction in older US-Americans but found no association with PA [125]. Oktaviana et al. conducted a longitudinal study in Indonesia measuring SOSU provision from living with a spouse and children and found no significant association between SOSU and PA [126]. Okoye et al. found a negative association between perceived SOSU and PA in older Nigerians [36]. Several studies have focused on the sources of SOSU but found inconclusive evidence. Van Cauwenberg et al. found a positive association between neighbourhood SOSU and walking [127] in older Belgians. Thornton et al. observed positive associations between objective and subjective PA and SOSU from family, friends, acquaintances and co-workers in older US-Americans [128]. In a longitudinal study of older US-American women, Harvey and Alexander found that SOSU from friends significantly predicted PA and had a positive effect on PA levels. However, SOSU from spouses or children did not show a significant effect [28]. Lian at el. examined the relationship between PA and SOSU from family and friends in Singapore and found that family SOSU was positively associated with PA [129]. Qu et al. found a significant positive association between group PA and SOSU from family and others but found no association with SOSU from friends in older Chinese [41]. Lack of SOSU from friends and neighbours was found to be significantly associated with lower levels of PA in older Japanese by Kanamori et al. [130]. Wang et al. analysed the relationship between SOSU from family and exercise in older Chinese and found a positive association [131].

Qualitative studies have also provided valuable insights. Rowland et al. identified family support and encouragement from friends as essential enablers of PA for Indigenous Australians [26]. Kegler et al. highlighted church communities as important sources of support in the USA [132]. Jones et al. highlighted emotional and instrumental SOSU from family, friends and fellow church members as essential enablers of PA in older US-American women [94]. In an US-American intervention study, Floegel et al. found that sufficiently active participants reported more SOSU from friends and family than insufficiently active participants [123]. Zhang et al. also identified family, partners and friends as important sources of SOSU to motivate exercise, although family responsibilities could be a barrier [124]. Another study in the USA found SOSU from friends, family and peers an important facilitator of older women’s participation in group classes [116]; elsewhere, SOSU from family and friends motivated older Spanish adults to engage in group exercise [81, 119]. The importance of trainers’ SOSU for older Canadian adults to participate in group PA classes was highlighted by Morrison et al. [133]. Gomes et al. analysed factors encouraging or discouraging exercise among older adults in 16 European countries using the SHARE: the study showed that participants who received help had lower levels of PA, whereas participants who gave help had higher levels of PA [32]. Sjöberg et al. examined the quality of SOSU and changes in PA during the COVID-19 pandemic in Sweden. They found that low levels of SOSU before the pandemic were associated with reductions in higher-intensity PA in older adults aged 80 and over [134]. Kim et al. [135] investigated the development of SOSU through PA in South Korea, finding that people who were members of a sports club created and maintained positive social interactions with other participants and developed close friendships. This finding relates to companionship SOSU and highlights the bidirectional relationship between PA and SOSU. Overall, these studies underscore the importance of different forms of SOSU in motivating and facilitating PA in older adults.

## Discussion

This paper aimed to update a systematic review [8] and deepen the existing understanding of our topic by encompassing both quantitative and qualitative research. The primary focus was to examine the relationship between functional SOSU and PA in older adults. This scoping review included a significant amount of new evidence, highlighting the salience of the topic. Notably, 39% of the studies reviewed were published between 2020 and August 2023. In general, it can be concluded that functional SOSU is a key factor in PA and exercise in older adults. These findings are consistent with the systematic review by Lindsay-Smith et al. [8] but also with reviews analysing the situation in adolescents [13, 136]. By including qualitative evidence, we gained deeper insights into the barriers and facilitators for engaging in physical activity among older adults. For instance, Lindsay Smith et al. also emphasised the significance of peer programmes [8]. The qualitative studies provided additional information on how these programmes contribute to PA adherence, such as increased feelings of safety and the opportunity to maintain social relationships with others. Comparing the quantitative results remains challenging due to the considerable variation in the measures used to assess both PA and SOSU across studies. However, when using comparable instruments, studies have shown a clearer association. For example, five studies used the PASE to assess PA and SSES to assess SOSU. Four studies reported a significant positive association [27, 30, 66, 69], while one study found no significant association [21]. Similarly, to the findings of Lindsay Smith et al., only SOSU for PA was often measured with the same instrument (SSES) whereas for the other types of SOSU the measurements varied so much that comparison is very difficult and therefore the results seem less reliable for other types of SOSU than for PA SOSU. This furthermore shows that using instruments that measure specific the PA of older adults might produce different outcomes in the association between PA and functional SOSU than when using not age-specific instruments. Similar to Lindsay Smith et al.‘s findings, only SOSU for PA was measured consistently using the SSES instrument. For other types of SOSU, the varying measurements make comparison difficult, resulting in less reliable results. Furthermore, this demonstrates that using instruments that measure the PA of older adults specifically may yield different results in the relationship between PA and functional SOSU compared to using non-age-specific instruments. When PA has been measured objectively, there has been conflicting evidence regarding its association with SOSU. Some studies reported a positive association [20, 44, 45, 63, 75, 128], whereas others [21, 61, 109] found no such association. Seguin-Fowler et al. even found a negative association between both objective and subjective PA and SOSU [37]. Furthermore, specific types of PA, such as park-based PA [108] or walking [24, 71, 105, 127], yielded different results from general PA.

For future research and public health interventions, it would be beneficial to use consistent measures for both PA and SOSU. Additionally, it would be helpful to use age-specific instruments for this study population, as older adults face different challenges in adhering to PA recommendations and their SOSU networks change due to ageing processes such as retirement or widowhood [137]. This is particularly important given the inconclusive evidence on sources of SOSU. As sources of SOSU change with age, it would be useful to have a tool that specifically addresses this issue. This scoping review found that several studies used instruments designed for an older population, such as CHAMPS or PASE, to assess PA. However, for SOSU, none of the studies used age-specific instruments.

Regarding our study population, it is logical that both companionship and informational support from SOSU are important facilitators of PA. This is because they increase the feeling of safety when performing PA. Professional informational SOSU is particularly crucial for exercise and PA in older adults, possibly due to concerns about exercise safety in later life. Therefore, this aspect should be carefully considered when designing PA interventions for older adults.

Instrumental SOSU on the other hand stands out in the proportion of quantitative studies reporting significant negative associations. This may be because people who require instrumental SOSU face greater challenges in engaging in PA due to lower levels of fitness and health. One barrier to PA is the lack of financial sources and financial instrumental SOSU could help to overcome this problem. As older adults often face challenging financial situations, this is an area that requires further attention. Instrumental SOSU can address another barrier to PA, which is when older adults act as caregivers for their spouses. Barriers include family responsibilities and lack of support [22, 27, 96, 104]. Conversely, one motivation for exercising was to maintain good health and avoid becoming a burden on family members [99, 124].

Future studies and public health interventions should also focus on minority groups, such as ethnic minorities or migrants, to address the barriers and facilitators for engaging in PA. Older ethnic minorities, in particular, are an understudied group in this area. For instance, migrants may have smaller or different SOSU networks and may lack information about low-cost community PA programmes. Additionally, cultural aspects of PA engagement should be considered when developing such programmes [29].

Overall, companionship SOSU contributes significantly to PA participation, especially in group-based programmes, and promotes social connections, ultimately benefiting the physical and social aspects of older adults’ lives. in turn, lead to increased PA. Safety is a critical issue to consider, and both companionship and informational SOSU can increase feelings of safety when engaging in PA. Difficulties related to safety, such as neighbourhood safety [22, 96] and safe exercise practices, can be significant barriers to PA in older adults.

The concept of functional SOSU relates to the idea that interpersonal relationships provide multiple different types of SOSU and that these types of SOSU may be more or less important and effective in addressing specific problems or challenges [12]. Regarding adherence to PA recommendations in older adults, it is clear that all five dimensions of functional SOSU are associated with adherence. Although the evidence is mixed, companionship SOSU in particular appears to be a key enabler of PA in older adults.

### Limitations and strengths

This scoping review has several limitations, particularly in relation to our inclusion criteria. We excluded studies that focused on patient groups; as a result, we may have ‘lost’ many pertinent articles. However, we decided to do this as patients are likely to have different SOSU needs and may face different challenges in engaging in PA. In this review, we defined older adults broadly as those aged 60 or over and did not consider age groups of older adults in our analysis. However, there are significant variations between young-old, mid-old, and old-old individuals and the possibility of engaging in PA or exercise varies among different age groups. In addition, we only included articles that were written in English. Thus, there is are limited information about culture factors, such as race and ethnicity, related to SOSU and PA among older adults. Finally, this review focuses only on functional SOSU. Therefore, it excludes any form of structural SOSU. In addition, as we only included studies dealing with SOSU, papers analysing related concepts such as social networks, social integration and loneliness were not included. There is also a significant degree of heterogeneity between studies in terms of research design, sample size and specific study populations, which makes it difficult to compare results. A further limitation of scoping reviews is that they do not assess the quality of the studies; moreover, as they include qualitative studies, the results cannot be easily compared or synthesised. However, the inclusion of qualitative studies is a major advantage of this paper as these provide a deeper insight into the research topic. The greater breadth of scoping reviews also allowed us to include more studies with different variables (e.g. not only general PA but also park-based PA). This scoping review is therefore an informative update to the 2017 systematic review. Though the results cannot be generalised, this review provides a comprehensive overview of all available evidence on the topic.

### Conclusion and implications

This review identifies which types of functional SOSU are associated with PA in older adults. There is considerable heterogeneity in the design of studies on this topic. While the evidence on the association between functional SOSU and PA is mixed, most studies show a positive association. In particular, SOSU from family, friends or an exercise group seems to increase PA. PA itself may also provide a strategy for improving social contact and social integration.

Further research is needed to not only assess but also improve PA in older adults. In particular, more longitudinal studies would be highly beneficial to this area of research to make conclusions about the direction of functional SOSU and PA. In addition, consistent use of validated scales could help in comparing results between studies. While some studies mentioned above used the CHAMPS or PASE, it would be helpful if future research used more age-specific measures; this also applies to SOSU instruments. It is clear that increasing SOSU for older people could have a positive effect on their PA levels. As PA is such an important aspect of healthy ageing, SOSU should be promoted in older adults. However, such public health interventions must be accessible to adults with lower SOSU from family and friends, for example, by involving GPs or other health professionals.

### Electronic supplementary material

Below is the link to the electronic supplementary material.

Supplementary Material 1

Supplementary Material 2

## Data Availability

All data generated and analysed for the review are available upon request from the corresponding author.

## Abbreviations

PA: Physical activity
SOSU: Social Support
IPAQ: International Physical Activity Questionnaire
CHAMPS: Community Healthy Activities Model Program for Seniors
PASE: Physical Activity Scale for the Elderly
SSES: Social Support for Exercise Scale
DSSI: Duke Social Support Index
MSPSS: Multidimensional Scale of Perceived Social Support
LSNS: Lubben Social Network Scale
