# Supplementary material for: Physical activity and functional social support in community-dwelling older adults: a scoping review
Source: BMC Public Health. 2024 May 20;24:1355. doi: 10.1186/s12889-024-18863-6 (PMC11103817; doi:10.1186/s12889-024-18863-6)
Supplement: Supplementary file 2 — Supplementary Material 2 [file 12889_2024_18863_MOESM2_ESM.pdf]

S1 Table: Data extraction table for the included studies

Data extraction table for cross-sectional studies and the quantitative elements of mixed methods studies

Authors	Year	Country	Age (years): m, [R] [a]	Gender	N[b]	Type of PA	PA Measure	Type of SOSU	SOSU measure	Res-ults [c]
Andrade de Sousa et al. [83]	2021	Brazil	69.9 [60–88]	44.7% female	208	Subjective PA: MVPA	IPAQ<br>PASE	PA SOSU	SSPAS	**+**
Bakhtar et al. [84]	2019	Iran	[60+]	not stated	340	Subjective PA: MVPA	PASE-SF, last 7 days)	PA SOSU	20 items (5 items assess friends to support and 15 items measure family support.)	**+**
Blakoe et al. [38]	2023	Spain	[65+]	60% female	7,023 (older age group)	Subjective PA: LTPA, MVPA	2 items: Doing PA in the leisure time at least several times a week; reported frequent PA during daily activity	General	DSSI	**+**
Bopp et al. [27]	2004	USA	70.6	100% female	102	Subjective PA: MVPA<br>Strength training	PASE<br>Strength training exercise	PA SOSU	SSES	**+**
Carlson et al. [63]	2012	USA	74.4	53.1% female	718	Objective PA<br><br>Subjective PA: MVPA	Accelerometer (1 week)<br>CHAMPS	PA SOSU	SSES (adapted, 4 items)	**+**
Chan et al. [39]	2020	Malaysia	[60+]	51% female	3,969	Subjective PA: MVPA	GPAQ	General	DSSI	**+**
Chan et al. [44]	2020	USA	74.1	52% female	400	Objective PA	Accelerometer (at least 7 consecutive days)	General	Lifestyle Questionnaire: 9 items regarding the availability of SOSU in various circumstances	**+**
Chen et al. [45]	2021	Sweden	[60+]	Age group < 70: 60.2% > 80: 69.9%	595	Objective PA	Accelerometer (at least 7 consecutive days)	General	9 items: Satisfaction with social contacts, instrumental SOSU, emotional SOSU, feeling of social embeddedness	**+**
Chen et al. [46]	2015	China	76.5 [60-99]	66% female	521	Subjective PA: LTPA, HPA, MVPA	Self-reported items on frequency of walking, MVPA, LTPA, HPA	General	SSRS	**+**

Corseuil Giehl et al. [71]	2017	Brazil	70.3	63.% female	1,705	Subjective PA: Walking	IPAQ (walking domain)	PA SOSU	Scale of Social Support for Physical Activity	**+**
Eronen et al. [85]	2012	Finland	77.6 [75–81]	75% female	629	Subjective PA: Unmet PA need	2 items: “Do you feel that you would have the opportunity to increase your level of physical activity if someone recommended you to do so?”; “Would you like to increase your level of physical activity?”	Emotional	1 item: Someone to talk to	**+**
Gomes et al. [32]	2017	Europe (SHARE)	67.8	59.2% female	19,298	Subjective PA: MVPA	2 items: PA frequency	Given and received help	2 items: Received help from others, given help last 12 months	*****
Gothe [21]	2018	USA	64.8 [55–75]	87.2% female	110	Objective PA:<br><br>Subjective PA: MVPA	Accelerometer (7 consecutive days)<br>PASE<br>GLTEQ	PA SOSU	SSES	**0**
Gyasi [50]	2019	Ghana	66	63% female	1,200	Subjective PA: MVPA	GPAQ: 1 item	General	2 items: Contact frequency; social participation	**+**
Hall & McAuley [64]	2010	USA	69.6	100% female	128	Objective PA: Daily steps	Accelerometer (7 days)	PA SOSU	SSES	**0**
Jin et al. [47]	2022	China	69	69.5% female	778	Subjective PA: MVPA	IPAQ-SF	General	SSRS	**+**
Kanamori et al. [130]	2023	Japan	75	59% female	1,494	Subjective PA: Exercise habits	1 item: Do you walk or do household chores (cleaning, gardening, etc.) that constitute exercise at least once per week?’	SOSU source	1 item: Who do you consults with about your problems?	**+**
Kang et al. [49]	2018	South Korea	[65+]	61% female	332	Subjective PA: LTPA	GLTEQ	General	SSQ	**+**
Kaplan et al. [52]	2001	Canada	[65+]	50.4% female	12,611	Subjective PA: LTPA	1 item: Monthly LTPA more than 15 minutes	General	4 items: Someone they could confine in; someone they could count on; someone who could give them advice; someone who made them feel loved	**+**
Kim & Kosma [65]	2012	South Korea	68.56 [65–89]	70% female	290	Subjective PA: LTPA	GLTEQ	PA SOSU	SSES (adapted)	**+**

Krause et al. [86]	1993	Japan	68.7	51% female	2,200	Subjective PA: MVPA	3 items on exercise, walking and gardening frequency	Emotional	2 items: Someone to talk to; feeling loved and cared	**+**
Kumar et al. [42]	2022	India	67.2 [60–95]	58% female	400	Subjective PA: Exercising	Items via Katz ADL	General	LSNS	**+**
Kwag et al. [53]	2011	USA	81.84 [66–106]	72.8% female	163	Subjective PA: MVPA	PASE	Perceived	SPS-SF	**+**
Lee & Fan [82]	2023	Taiwan	71.41 (older age group)	68.36% female	183 (older age group)	Subjective PA: MVPA	PASE	PA SOSU	5 items: Assessing SOSU from family and friends for PA, such as engaging in PA with the participant or encouraging them to take PA	+
Levy-Storms & Lubben [23]	2006	USA	61 [50–97]	100% women	290	Subjective PA: Exercising	1 item: Exercises at all	General	LSNS	**+**
Lian et al. [129]	1999	Singapur	[60–80+]	58% female	2,494	Subjective PA: MVPA	Weekly frequency of MVPA	SOSU source	Rewards and SOSU from family members + friends	**+**
Loprinzi & Crush [92]	2017	USA	71.4, 60-85	50.7% male	2,519	Objective PA	Accelerometer (at least 4 consecutive days)	Emotional<br><br>Instrumental	2 items: Provision of emotional support in the last 12 months<br><br>1 item: Having someone to help financially if needed	**+**
Loprinzi & Joyhner [87]	2016	USA	71.4	50.1% female	5,616	Subjective PA: LTPA	Question about 48 different activities in the last 30 days (16 sport, 14 exercise and 18 recreational activities)	Emotional<br><br>Instrumental	2 items: Provision of emotional support in the last 12 months<br><br>1 item: Having someone to help financially if needed	**+**
Malek Rivan et al. [35]	2022	Malaysia	71.18	55% female	535	Subjective PA: MVPA	IPAQ-SF	Emotional Instrumental	Items based on the Brief COPE inventory	**-**
Marthammuthu et al. [33]	2023	Malaysia	70.83	100% female	1,221	Subjective PA: MVPA	PASE	General	DSSI	*****
Morris et al. [51]	2010	Jamaica	[55–64] [65–74] [75+]	100% male	2,000	Subjective PA: Exercising	2 items	General	1 item: Having SOSU (yes/ no)	**+**
Mowen et al. [125]	2007	USA	67.4 [55-99]	66% female	1,515	Subjective PA: Daily PA levels	1 item: Daily PA levels	General	SSQ	**0**
Mudrak et al. [66]	2017	Czech Republic	68	79.2% female	546	Subjective PA: LTPA MVPA	GLTEQ PASE	PA SOSU	SSES	**+**

Newsom et al. [78]	2018	USA	72.55	58.5% female	217	Subjective PA: MVPA	CHAMPS	PA SOSU	16 items	**+**
Niedermeier et al. [57]	2019	Germany	70 [60–80]	50% female	100	Subjective PA: MVPA	PAQ-50+	General	SSQ	**+**
O'Brien Cousins [79]	1995	Canada	77 [70–98]	100% female	327	Subjective PA: Exercising	OA-ESI	PA SOSU	4 items covering family athletism, encouragement, PA activity levels of close contacts	**+**
O'Brien Cousins [80]	1996	Canada	76.7 [70–98]	100% female	327	Subjective PA: Exercising	OA-ESI	PA SOSU	4 items covering family athletism, encouragement, PA activity levels of close contacts	**+**
Oh et al. [40]	2020	South Korea	[50–65]	44.3% female	778	Subjective PA: MVPA	IPAQ	General	MSPSS	**+**
Oka & Shibata [76]	2012	Japan	74.5 [70–89]	53% female	137	Objective PA:	Accelerometer: Daily number of steps. Recorded over 1 year and data downloaded every month	PA SOSU	5 items: functional, emotional + informational PA SOSU	**0**
Okoye et al. [36]	2022	Nigeria	74.25	65% female	100	Subjective PA: MVPA	PASE	SOSU source	MSPSS	**-**
Orsega-Smith et al. [67]	2003	USA	69 [60–88]	80% female	265	Objective PA: Participation level in an exercise programme	Attendance	PA SOSU	SSES	**+**
Orsega-Smith et al. [68]	2007	USA	67.7	61.5% female	1,900	Subjective PA: LTPA	2 items: Type of LTPA; frequency over the last week	PA SOSU	SSES	**+**
Ory et al. [105]	2016	USA	69 [60–92]	50.37% female	272	Subjective PA: Walking	Walking (1 item)	Companionship	SOSU for walking (2 items)	**+**
Park et al. [69]	2014	South Korea	71.62	70.1% female	187	Subjective PA: MVPA	PASE	PA SOSU	SSES	**+**
Perrino et al. [24]	2011	USA	79.95 [72–102]	62% female	217	Subjective PA: Walking	Walking route in the last 7 days, transformed into "total blocks walked"	General<br>Emotional<br>Informational<br>Instrumental	Emotional (4 items)<br>Informational (4 items)<br>Instrumental (2 items)	**-**
Potts et al. [43]	1992	USA	72.57	59.6% female	936	Subjective PA: MVPA	Exercising adherence (frequency) in the last week	General	LSNS	**+**
Purath et al. [74]	2009	USA	67.4 [60–87]	62% female	34	Subjective PA: MVPA	PASE	PA SOSU	Social Support for Physical Activity Scale	**+**
Qu et al. [41]	2023	China	60.62	95.2% female	2,721	Subjective PA: Group exercising	PARS-3	General	MSPSS	**+**

Reed et al. [54]	2011	USA	[60+]	not stated	601	Subjective PA: LTPA	Frequency of LTPA in the last months	General	4 items on social contacts in a typical week	**+**
Riffle et al. [58]	1989	USA	74 [56–94]	78% female	113	Subjective PA: Exercising	1 item: Exercises at all	General	PRQ 2	**0**
Ryu et al. [60]	2023	USA	78.1 [63–99]	100% female	4,168	Objective PA	Accelerometer (7 days)	General	MOS	**0**
Salvador et al. [107]	2009	Brazil	[60–74]	60.5% female	385	Subjective PA: MVPA	IPAQ	Companionship	NEWS	**+**
Shores et al. [106]	2009	USA	[65–74] [75–84] [85+]	46.8% female	454	Subjective PA: Exercising	Exercise frequency (last 7 days)	Companionship	1 item: "I don't know anyone who would participate with me."	**+**
Sjöberg et al. [134]	2022	Sweden	[65–99] Age group ≤70: 66.1 Age group ≥80: 84	Age group ≤70: 64.8% female Age group ≥80: 67.9% female	624	Subjective PA: Change of MVPA levels during the pandemic as compared to pre-pandemic levels	Self-reported changes of LVPA + MVPA	Quality of SOSU	4 items: Reported satisfaction with the social contacts; perceived material and psychological support; sense of affinity with association members, relatives, and living area; being part of a group of friends	**+**
Thornton et al. [128]	2017	USA	74.4	53% female	726	Objective PA:<br><br>Subjective PA: MVPA	Accelerometer (7 consecutive days)<br>CHAMPS	PA SOSU	4 items: How often during the past 6 months their family, friends, acquaintances or co-workers (1) walked or exercised with them, (2) gave them encouragement to do physical activity, (3) made positive comments about the participant's physical appearance, and (4) criticized or made fun of them for walking or exercising	**+**
Umstattd et al. [59]	2006	USA	70.4	78% women	296	Subjective PA: MVPA	CHAMPS-SF	PA SOSU	PASS	**0**
Van Cauwenberg et al. [127]	2014	Belgium	74.3	55.6% female	50,986	Subjective PA: Walking	1 item: Walking for transportation	Neighbours SOSU	1 item: Counting on help from neighbours	**+**

Van Luchene et al. [62]	2021	Belgium	67.59 sub-group retirees	74.36% female (sub-group)	39 (sub-group)	Subjective PA: MVPA	SOC, IPAQ-SF	PA SOSU	SSES	**+**
Wagner et al. [108]	2020	Germany & China	71.03	52.7% female	617	Subjective PA: Park-based PA	3 items: Type, amount and intensity levels of PA in parks during a typical week	Companionship	3 items	**0**
Wang et al. [131]	2022	China	[60+]	41% female	7,901	Subjective PA: Exercising	1 item: Exercise activities: went to a sport, social, or other kind of club in the past months (based on CHARLS)	SOSU source	5 items: Marital status; number of children; frequency of meeting with their children, whether their children provide financial support; whether they are satisfied with their relationship with their children	**+**
Watt et al. [88]	2014	USA	71.7	55.6 % female	4,014	Subjective PA: MVPA	1 item: PA in the last 30 days	Emotional	2 items: Provision of emotional support in the last 12 months	**+**
Wendt Böhm et al. [77]	2016	Brazil	69.5	63.4% female	1,285	Subjective PA: LTPA	IPAQ (leisure domain)	PA SOSU	PASSS	**+**
Wilcox et al. [30]	2003	USA	70.6	100% female	102	Subjective PA: MVPA	PASE	PA SOSU	SSES	**+**
Wilcox et al. [55]	2000	USA	67.43	100% female	2,912	Subjective PA: Exercising	Items adapted from the NHIS + the BRFSS about frequency and intensity of exercising in the last 14 days	PA SOSU	4 items	**+**
Yamakita et al. [91]	2015	Japan	73.5	51.6% female	78,002	Subjective PA: Participation in sports groups	1 item: Frequency of participation in sports groups	Emotional Instrumental	4 items: Emotional + instrumental SOSU received + given (yes/no)	**+**
Yi et al. [48]	2016	China	67.11 [60–85]	63.3% female	1,580	Subjective PA: Exercising	3 items: Exercise frequency + duration	General	5 items assessing emotional and informational SOSU	**+**
Zimmer & McDonough [34]	2021	Canada	72.78 [65–89]	53.4% female	21,491	Subjective PA: MVPA	PASE	General Instrumental	Items on instrumental support, affectionate support, emotional/ informational support + positive social interaction	*****

									(items based on the MOS)	

[a] m = mean, [R] = range
[b] N = sample size
[c] Results: **0** indicates no sig. relationship ($p \geq 0.05$), **+** indicates sig. pos. relationship ($p < 0.05$), **-** indicates sig. neg. relationship, ***** indicates sig. mixed relationship
Abbreviations: PA = physical activity; SOSU = social support; MVPA = moderate-vigorous PA; LTPA = leisure-time PA; HPA = household PA

Physical activity measures: CHAMPS - Community Healthy Activities Model Program for Seniors; GLTEQ – Godin's Leisure Time Exercise Questionnaire; GPAQ - Global Physical Activity Questionnaire; IPAQ – International Physical Activity Questionnaire; PASE - Physical Activity Scale for the Elderly; PAQ+50+ - German Physical Activity Questionnaire 50+; SOC - Stage of Change to Exercise Behaviour Scale; OA-ESI - Older Adult Exercise Status Inventory; Katz ADL - Activities of Daily Living Index; PARS-3 - Physical Activity Rating Scale-3; SBQ - Sedentary Behavior Questionnaire

Social support measures: SSPAS **-** Social Support for Physical Activity Scale; SSES – Social Support for Exercise Scale; DSSI - Duke Social Support Index; SSRS - Social Support Rate Scale; SSQ - Perceived Social Support Questionnaire; SPS - Social Provision Scale; LSNS - Lubben Social Network Scale; PRQ 2 - Personal Resource Questionnaire; NEWS - Neighborhood Environment Walkability Scale; PASSS - Physical Activity Social Support Scale; PASS – PA Social Support; MSPSS - Multidimensional Scale of Perceived Social Support; OSSS-3 - Oslo-3 Social Support Scale; MOS – Medical Outcomes Study

Data extraction table for observational longitudinal studies

Authors	Year	Country	Age (years): m, [R] [a]	Gender	N[b]	Type of PA	PA Measure	Type of SOSU	SOSU measure	Results [c]
Harvey & Alexander [28]	2012	USA	69	100% female	671	Subjective PA: MVPA	3 items: "How often they engaged in the following activities: working in the garden or yard, participating in active sports or exercise, and taking walks"	SOSU source	3 items: SOSU from spouse; friends; children	**+**
Komazawa et al. [90]	2021	Japan	68.9	56.6% female	3,911	Subjective PA: Exercising	1 item: Exercise frequency in general	Emotional<br><br>Instrumental	2 items: "How often does someone listen to you?" and "How often does someone show you love and understanding?"<br><br>2 items: "How often does someone care for you when you are sick?" and "How often does someone provide financial assistance?"	**0**
Manz et al. [89]	2018	Germany	60 [50–78]	52.5% female	1,184	Subjective PA: LTPA	1 item: Frequency of LTPA	Emotional	1 item from OSSS-3	**+**

Oktaviani et al. [126]	2022	Indonesia	[60–70+]	52% female	1,289	Subjective PA: MVPA	IPAQ	Having SOSU	1 item: Living with spouse and children (yes/ no)	**0**
Smith et al. [72]	2023	Australia	61.7 [60–65]	61.4% female	1,984	Subjective PA: LTPA/ MVPA	Items assessing the frequency + duration of LTPA + MVPA in the last week (using items from the Active Australia Survey)	PA SOSU	5 items: Rate how often family or friends provided each of different types of SSPA (emotional, instrumental, informational and companionship) over the last three months	+
Warner et al. [70]	2011	Germany	65+ [65–89]	42% female	309	Subjective PA: Exercising	1 item: Exercise frequency in the last 7 days	SOSU for PA	SSES-SF	**+**

[a] m = mean, [R] = range at baseline
[b] N = sample size at baseline
[c] Results: **0** indicates no sig. relationship ($p \geq 0.05$), **+** indicates sig. pos. relationship ($p < 0.05$), **-** indicates sig. neg. relationship, ***** indicates sig. mixed relationship
Abbreviations: PA = physical activity; SOSU = social support; MVPA = moderate-vigorous PA; LTPA = leisure-time PA

Physical activity measures: CHAMPS - Community Healthy Activities Model Program for Seniors; GLTEQ – Godin's Leisure Time Exercise Questionnaire; GPAQ - Global Physical Activity Questionnaire; IPAQ – International Physical Activity Questionnaire; PASE - Physical Activity Scale for the Elderly; PAQ+50+ - German Physical Activity Questionnaire 50+; SOC - Stage of Change to Exercise Behaviour Scale; OA-ESI - Older Adult Exercise Status Inventory; Katz ADL - Activities of Daily Living Index; PARS-3 - Physical Activity Rating Scale-3; SBQ - Sedentary Behavior Questionnaire

Social support measures: SSPAS **-** Social Support for Physical Activity Scale; SSES – Social Support for Exercise Scale; DSSI - Duke Social Support Index; SSRS - Social Support Rate Scale; SSQ - Perceived Social Support Questionnaire; SPS - Social Provision Scale; LSNS - Lubben Social Network Scale; PRQ 2 - Personal Resource Questionnaire; NEWS - Neighborhood Environment Walkability Scale; PASSS - Physical Activity Social Support Scale; PASS – PA Social Support; MSPSS - Multidimensional Scale of Perceived Social Support; OSSS-3 - Oslo-3 Social Support Scale; MOS – Medical Outcomes Study

Data extraction table for experimental studies

Authors	Year	Country	Age (years): m, [R] [a]	Gender	N[b]	Type of PA	PA Measure	Type of SOSU	SOSU measure	Res-ults [c]
Aguiñaga et al. [20]	2021	USA	67	75% female	20	Objective PA Subjective PA: LTPA, MVPA	Accelerometer CHAMPS SBQ	PA SOSU	SSES	+
Brassington et al. [61]	2002	USA	70.18	65% female	103	Objective PA: Exercising	Attendance	PA SOSU	SSES	0
Cai et al. [111]	2022	China	66.9	64% female	72	Objective PA: Daily walking steps	Acceleometer for 3 consecutive days	Companionship	Peer support intervention arm	+
Chia et al. [113]	2023	Taiwan	71.6 (male) 72.3 (female)	50% female	120	Objective PA: Steps	Accelerometer	Companionship	6 items from the Health-Promoting-Lifestyle-Scale: "Discuss exercise patterns with friends"; "I would compliment a	+

									friend’s athleticism”; “Exercise with friends”; “I will show concern to my friends”; “Playing sports with friends makes me happy”; “get support from friends while exercising”	
Crist et al. [112]	2022	USA	71	76% female	476	Objective PA	Accelerometer	Companionship	Peer-led intervention, group walks	+
Gellert et al. [31]	2011	Germany	66.5	48% female	302	Subjective PA: HPA, LTPA/ sport/ TPA	PAQ-50	PA SOSU	SSES (adapted, 2 items)	*
Huang et al. [114]	2022	Taiwan	70.8 (arm 1) 71 (arm 2) 71.5 (arm 3)	100% male	60	Objective PA: Steps + walked distance Subjective PA: MVPA, LTPA	Accelerometer<br><br>6 items: VPA for at least 3 times for 30 minutes a week; MPA for at least 5 times a week for 30-40 minutes; stretching; LTPA; HPA; pulse checking	Companionship	Arm 1: Walking together in the park for at 30 minutes from Monday to Friday	+
McAuley et al. [109]	2003	USA	66 [60–75]	not stated	153	Objective PA: Exercising Subjective PA: LTPA	Attendance<br><br>PASE	General Companionship	SPS<br>Group exercise	0
Ory et al. [75]	2018	USA	74.8 (arm 1) 74.3 (arm 2)	79.1% (arm 1) 76.4% (arm 2)	430	Objective PA: Exercising Subjective PA: MVPA	Attendance<br><br>IPAQ	PA SOSU	4 items: How frequently receiving SOSU for planning PA goals; keeping PA goals; reducing barriers to PA; engaging in PA	+
Rhodes et al. [73]	2001	Canada	76.4 [75–80]	100% female	30	Objective PA: Exercising	Attendance	PA SOSU	1 item: "The people I spend most of my time with now encourage me in physical fitness activities."	+
Seguin-Fowler et al. [37]	2021	USA	64.8 (arm 1) 65.1 (arm 2)	80.7% female (arm 1) 75% female (arm 2)	167	Objective PA: All<br><br>Subjective PA: LTPA, MVPA	Accelerometer (7 consecutive days)<br>CHAMPS<br>IPAQ	PA SOSU	SSES	-
Thomas et al. [110]	2012	China	72.4 (arm 1)	67% female (arm 1)	399	Subjective PA: MVPA	IPAQ	Companionship	Peer buddy support arm	+

			71.7 (arm 2)	65.3% female (arm 2)						
Wilcox et al. [56]	2008	USA	[50+]	79.1% female (arm 1) 82.5% female (arm 2)	1335	Subjective PA: LTPA	CHAMPS	General	5 items (developed from the US Women's Determinants Study)	+

[a] m = mean, [R] = range
[b] N = sample size
[c] Results: **0** indicates no sig. relationship ($p \geq 0.05$), **+** indicates sig. pos. relationship ($p < 0.05$), **-** indicates sig. neg. relationship, ***** indicates sig. mixed relationship
Abbreviations: PA = physical activity; SOSU = social support; MVPA = moderate-vigorous PA; LTPA = leisure-time PA; HPA = household PA; arm = study arm

Physical activity measures: CHAMPS - Community Healthy Activities Model Program for Seniors; GLTEQ – Godin's Leisure Time Exercise Questionnaire; GPAQ - Global Physical Activity Questionnaire; IPAQ – International Physical Activity Questionnaire; PASE - Physical Activity Scale for the Elderly; PAQ+50+ - German Physical Activity Questionnaire 50+; SOC - Stage of Change to Exercise Behaviour Scale; OA-ESI - Older Adult Exercise Status Inventory; Katz ADL - Activities of Daily Living Index; PARS-3 - Physical Activity Rating Scale-3; SBQ - Sedentary Behavior Questionnaire

Social support measures: SSPAS **-** Social Support for Physical Activity Scale; SSES – Social Support for Exercise Scale; DSSI - Duke Social Support Index; SSRS - Social Support Rate Scale; SSQ - Perceived Social Support Questionnaire; SPS - Social Provision Scale; LSNS - Lubben Social Network Scale; PRQ 2 - Personal Resource Questionnaire; NEWS - Neighborhood Environment Walkability Scale; PASSS - Physical Activity Social Support Scale; PASS – PA Social Support; MSPSS - Multidimensional Scale of Perceived Social Support; OSSS-3 - Oslo-3 Social Support Scale; MOS – Medical Outcomes Study

Data extraction table for qualitative studies and the qualitative elements of mixed methods studies

Authors	Year	Country	Age (years): m, [R] [a]	Gender	N[b]	Type of PA	Type of SOSU	Results
Arnautovska et al. [101]	2017	Australia	73.3	64.6% female	48	Motivation to engage in PA	Informational General Instrumental	Informational SOSU from health care professionals, general SOSU and instrumental SOSU were important facilitators for PA.
Beselt et al. [102]	2023	Canada	66.9 [57–79]	100% female	16	Group PA classes	Companionship Informational Validation	(1) Exercising together was seen as safer than exercising alone, particularly because of health concerns and fears of falling, and informational SOSU from the instructor was very important for safe training. (2) The positive obligation to attend stemmed from the opportunity to socialise with other participants, and participants valued the mutual accountability that led them to check on each other if someone had missed a class. (3) Some participants found it motivating to compare themselves with other participants and were inspired by seeing other women engaged in PA; observing women with lower fitness levels also motivated participants to maintain or increase their own levels of PA.

Bidonde et al. [115]	2009	Canada	75 [67–83]	100% female	9	Group exercise programme	Companionship	Through the programme, participants formed relationships and bonded with other women in similar positions. Friendships emerged outside the programme.
Bopp et al. [27]	2004	USA	67.5	100% female	39	Strength training	General	Lack of social support and family obligations were barriers to training.
Choi et al. [122]	2018	USA	69 [55–79]	100% female	64	Team sport	Companionship	(1) Friendship and social connections were important motivators for players to join the team and continue playing.<br>(2) Participation strengthened social connections with others; through the sport, participants developed a social network.
Choi et al. [103]	2022	South Korea	77 [66–88]	66.6% female	15	Exercising	Instrumental (financial)<br>Companionship	(1) Financial support was an important facilitator of exercise, given the importance of limited resources as a barrier to PA.<br>(2) Participants preferred group exercise because of the opportunity for social interaction and increased motivation.
Du et al. [116]	2023	USA	69.2	100% female	13	Group exercise programme	Companionship<br>SOSU source	(1) Peer support within the class played an important role in maintaining PA engagement.<br>(2) Support from friends, family and peers was key to participation in group exercise.
Floegel et al. [123]	2015	USA	65	87.5% female	24	Peer-led advice and support PA group	Companionship<br>SOSU source	(1) SOSU within the peer group and from mentors was a facilitator of PA engagement for both active and insufficiently active participants.<br>(2) More active participants reported greater SOSU from family and friends, even outside the intervention context, than less active participants.
Gagliardi et al. [100]	2020	Italy	75.7	42% female	13	Group exercise programme	Emotional<br>Informational<br>Companionship<br>Validation	Emotional, informational and companionship SOSU and validation were important motivators and facilitators for PA.
Gayman et al. [117]	2022	Canada	64 [55–73]	17.6% female	17	Team sport	Companionship	Exercising in a team sport was perceived as very positive as the team was seen as an important source of SOSU and increased participants’ social network.
Gothe & Kendall [22]	2016	USA	63 [55–75]	100% female	20	LTPA	General<br>Emotional<br>Companionship<br>Informational	(1) SOSU, specifically emotional and companionship SOSU, was considered one of the most important motivators for LTPA.<br>(2) Most participants preferred group exercise programmes; giving SOSU was also important.<br>(3) Participants valued informational support from physicians.<br>(4) Peer pressure, family responsibilities and poor neighbourhood conditions were considered barriers to exercising.
Horne et al. [25]	2012	UK	[60–70]	50% female	46	Taking up and maintaining PA	Emotional<br>Companionship	(1) In taking up PA, encouragement and motivation from family, friends and peers but also statutory and voluntary workers was crucial. SOSU made participants more confident in starting PA and was the most important factor in PA adherence.<br>(2) Exercising in a group was important because of the socialising opportunities.
Huffman et al. [93]	2021	USA	76	68.4% female	38	PA maintenance	Emotional<br>Informational<br>Validation	(1) For sustained PA engagement, informational and companionship SOSU were important facilitators.<br>(2) For re-engagement in PA, emotional SOSU from family and friends was especially important.

								(3) For the general maintenance of PA, validation (feedback) and informational and emotional SOSU were particularly important.
Janevic & Connell [104]	2008	USA	66.1	100% female	30	Exercising	Instrumental Companionship	Enablers for PA were having an exercise partner and instrumental SOSU, in particular having someone to watch the participant's care-receiver.
Jones et al. [94]	2020	USA	68	100% female	15	Exercising	Emotional Instrumental Companionship	(1) Emotional and instrumental SOSU provided by family, friends and fellow church members and companionship SOSU were important facilitators for PA.<br>(2) Group exercise provided opportunities to meet people and form new friendships, which was also an important facilitator.
Kegler et al. [132]	2010	USA	63 [50–70]	47% female	60	Exercising	SOSU source	SOSU from church friends constituted important support for exercising.
Kim et al. [135]	2014	South Korea	71 [66–83]	70% female	10	LTPA in a sports club	Development of SOSU	Participants emphasised the development of SOSU: most stated that by participating in various sports club activities, they created and maintained positive social interactions with other participants and established close friendships.
Kosteli et al. [118]	2016	UK	64 [54–79]	54% female	37	Exercising	Companionship	(1) Older adults specifically engaged in PA for social reasons: PA was considered crucial to social connections.<br>(2) Participants reported that having an exercise companion made them more motivated to engage in PA, while the lack of an exercise partner was a barrier.
Leung et al. [95]	2021	Hong Kong	72.8	50% female	38	Walking	Emotional Informational Companionship	(1) Being encouraged to walk by family, having friends to accompany them and having professional guidance encouraged participants to walk more.<br>(2) The majority of participants valued the increased sense of security and enjoyment of social interaction provided by walking in a group and preferred this to solo walking.
Marthammuthu et al. [96]	2021	Malaysia	74 [60–90]	100% female	17	Exercising	Emotional Instrumental Informational Companionship	(1) Informational, companionship and emotional SOSU were key factors encouraging exercise, while a lack of SOSU was a demotivator.<br>(2) The main barriers to PA were loss of companionship support, lack of instrumental support, logistical problems in attending exercise programmes and neighbourhood safety issues.
Martín-Moya et al. [119]	2022	Spain	68	77% female	39	Group exercise programme	Companionship SOSU source	(1) Group exercise provided opportunities to socialise and develop new friendships.<br>(2) SOSU from family and friends motivated participants to engage in group exercise.
Mathews et al. [29]	2010	USA	71	75% female	396	Exercising	Informational Instrumental	(1) Important barriers were lack of knowledge in terms of safety instructions and financial costs.<br>(2) General and senior-specific SOSU were important enablers.
Mobiliy et al. [120]	2017	USA	67 [54–81]	100% female	7	Group exercise programme	Companionship	SOSU and social interaction with other participants were very important and motivated participants to continue to exercise.
Morrison et al. [133]	2022	Canada	Phase 2: 69.49 [55–80]	Phase 2: 76.3% female	Phase 2: 38	Group exercise programme	SOSU source	Trainers supported autonomous PA engagement by encouraging and validating participants' perspectives and experiences, while trainers' knowledge and safe guidance increased participants' confidence and enabled them to complete the course.

Oliveria et al. [97]	2019	Australia	67<br>[54–84]	87% female	93	Exercise programme	Emotional<br>Information<br>Companionship	(1) Emotional and informational SOSU from family, friends and trainers were important facilitators.<br>(2) Participants valued the programme as a group-based activity with people of the same age, and they valued the interaction with peers and the opportunity to socialise outside the exercise context.
Orsega-Smith et al. [67]	2003	USA	69<br>[60–88]	80% female	50	Community-based exercise programme	Companionship	The exercise programme developed and promoted social support through interaction with peers and instructors who were in a similar age group.
Patterson et al. [98]	2022	Canada	71.6<br>[65–84]	100% female	14	Group exercise programme	Validation<br>Companionship	(1) Social comparison with other participants increased participants' motivation to engage in a group PA programme.<br>(2) Encouragement from other participants was an important factor in engagement.
Rowland et al. [26]	2021	Australia	[55–70]	100% female	7	PA	SOSU source	Family support and encouragement from friends were identified as important facilitators of PA, while family breakdown or separation and lack of support from friends and family were important barriers.
Victor et al. [121]	2016	UK	68<br>[61–75]	60% female	30	Walking	Companionship	The majority of participants who increased their walking level either participated in the trial as a couple or had someone else to walk with. Participants who did not improve their walking reported that they lacked SOSU and that this was a barrier.
Wahlich et al. [99]	2017	UK	[60–75]	62% female	60	PA maintenance	Emotional<br>Companionship	(1) Having friends and family to motivate participants to engage in PA was important; this support could be given either by exercising together or by giving participants the encouragement to exercise and try new exercise activities. Some participants mentioned that they exercised to stay healthy for their families.<br>(2) PA allowed participants to meet new people.<br>(3) Having no one with whom to engage in activity was perceived as an obstacle, with participants saying that a lack of SOSU caused them to walk less.
Zhang et al. [124]	2022	UK	78<br>[74–83]	47% female	92	Exercising	Companionship<br>Validation<br>SOSU source	(1) Companionship SOSU was important for exercising. Validation by social comparison was a further motivator.<br>(2) Family, partners and friends were considered important sources of SOSU and motivated participants to exercise. Participants also mentioned that they were motivated to exercise in order to stay healthy so as not to become a burden for their families. For people living alone, SOSU from friends was particularly important for encouraging exercise.<br>(3) Family responsibilities as a carer could be a barrier to exercising.
Zimmer et al. [81]	2023	Canada	Phase 2: 69.49<br>[55–80]	76.3% female	Phase 2: 38 (FGs)	Group exercise programme	Emotional<br>Companionship<br>Validation	(1) Participants valued the opportunity to make new social connections through group activities.<br>(2) The involvement of family or friends served as an important motivation to engage in PA. SOSU for PA from family, friends and others was very important as they provided instrumental, informational and emotional SOSU.<br>(3) Participants maintained a sense of security by checking on each other when someone missed a class.

								(4) Sharing feedback was an important motivator for group participation. Observing others performing exercises encouraged participants to try them themselves.

[a] m = mean, [R] = range
[b] N = sample size
Abbreviations: PA = physical activity; SOSU = social support; MVPA = moderate-vigorous PA; LTPA = leisure-time PA
